# Apoptosis Is a Demanding Selective Tool During the Development of Fetal Male Germ Cells

**DOI:** 10.3389/fcell.2018.00065

**Published:** 2018-06-28

**Authors:** Ignacio Bejarano, Ana B. Rodríguez, José A. Pariente

**Affiliations:** Neuroimmunophysiology and Chrononutrition Research Group, Department of Physiology, Faculty of Science, University of Extremadura, Badajoz, Spain

**Keywords:** primordial germ cells, gonocytes, spermatogonia, apoptosis, differentiation

## Abstract

Apoptosis is widely known to play a major role on diseases related to male infertility. Diseases of the male genital tract as defective spermatogenesis, decreased sperm motility, sperm DNA fragmentation, testicular torsion, varicocele and immunological infertility are strongly related to apoptotic cell death. Apoptosis must not be considered only as a fail on germ cell physiology or a secondary effect of certain pathologies and exogenous hazardous agents. Apoptosis orchestrates correct function and development of the male germ cell from the early embryonic stages of gonadal differentiation to the fertilization. In this review we have tried to address a reading frame of the main knowledge about apoptosis in male germ cell development. Focussing on mechanisms concerning cellular apoptosis, which are independent of exogenous stimuli, we aimed to highlight that apoptosis is a selective instrument that guarantees the delivery of genetic message to offspring.

## Introduction

Apoptosis is a physiological mechanism of programmed cell death, which entails the genetically determined elimination of misplaced or damaged cells. The proper functioning of the molecular mechanisms involved in apoptosis are essential for the maintenance, formation and restoration of tissues. Apoptosis induces cell death with high specificity and efficiency, guaranteeing the correct development and protection of organisms from a wide range of developmental abnormalities and diseases. On the contrary, the excess of apoptosis plays a pivotal role in the etiology of many pathologies, for instance in neurodegenerative diseases or cases of infertility (Espino et al., 2009; Monllor et al., 2017).

Apoptosis entails a genetic program that controls, designs and initiates a cascade of events that leads the cell forward an organized and noiseless destruction. That is to say, it does not initiate inflammatory response and it is ignored by the tissues around. One the main components of the apoptotic apparatus involves caspases (cysteinyl aspartate-specific proteases), a family of aspartic acid-directed cysteine proteases. Numerous proteins are target of caspases breaking down cellular structure and functions, which result in cell death (Stennicke and Salvesen, 1997). Two major apoptotic pathways have been traditionally described. One the one hand, the extrinsic or physiological pathway, triggered by extracellular physiological ligands, such as FasL which binds to its specific cell-surface death receptor (Ashkenazi and Dixit, 1998). On the other hand, the intrinsic pathway, mediated by mitochondrial signaling, in which several proteins are released from the mitochondrial intermembrane space into the cytoplasm (Green and Reed, 1998). Cytochrome *c*, which is included among the main initiator factors of apoptosis, mediates the activation of caspase-9 (Li P. et al., 1997) promoting the chain activation of caspases, including caspase-3 which leads cellular suicide. In this regard, Bcl-2 is a family of regulator proteins involved in the modulation of mitochondrial permeability and consequently in the regulation of apoptosis. Additionally, Bid, a Bcl-2 family member, performs the crosslinking between extrinsic and intrinsic apoptotic pathways. Upon stimulation of Fas receptors, caspase 8 activation takes place inducing a rapid activation of Bid and promoting its relocation to mitochondria as tBid. tBid directly competes with soluble Bcl-xL for binding Bax, thereby preventing the Bcl-xL-Bax interactions. This heterodimer favors their the Bax anchoring to the mitochondrial membrane, which is required for the formation of the mitochondrial apoptosis-induced channel (MAC) in the outer membrane. The permeabilization of outer mitochondrial membrane is needed for the release of cytochrome *c* among other pro-apoptotic factors (Kandasamy et al., 2003; Tan et al., 2005; Lovell et al., 2008).

Given the low transcriptional activity in spermatozoa, the presence of apoptosis was early denied in human ejaculated (Weil et al., 1998). However, nowadays the presence and activation of apoptotic signals in human spermatozoa in response to various stimuli is widely accepted (Eley et al., 2005; Barroso et al., 2006; Bejarano et al., 2008; Espino et al., 2009; Monllor et al., 2017). Several studies have reported that males suffering infertility from unknown origin, increased DNA damage and a subsequent fragmentation oligonucleosomal in sperm cells (Rex et al., 2017). Therefore, in line with this, growing evidences suggest that caspase activity is associated with spermatozoa immaturity, low count, reduced motility (Marchetti et al., 2004; Lozano et al., 2009), decreased fertilization rates (Grunewald et al., 2009) and loss of plasma membrane integrity, as shown by phosphatidylserine externalization (Paasch et al., 2004). Caspases are involved in andrological pathologies such as varicocele, immunological infertility and reduced sperm fertilizing potential (Said et al., 2006). Early works reported the presence of apoptosis in germ cells of males afflicted with severe azoospermia and oligozoospermia (Lin et al., 1997).

Likewise, the mitochondrial membrane potential (ΔΨ_m_) has been reported as a trustable tool to determine the sperm quality. Studies have observed a relationship between ΔΨ_m_ and events such as the integrity of plasma membrane permeability (Troiano et al., 1998), DNA damages (Donnelly et al., 2000), motility and *in vitro* fertilization rates (Marchetti et al., 2004) or even externalization of phosphatidylserine (Barroso et al. 2006). Similarly, spermatozoa showing high ΔΨ_m_ have shown intact functionality of acrosome as well as high motility values and fertilizing capacity (Gallon et al., 2006). On the contrary, low ΔΨ_m_ is related to low rates of pregnancy (Marchetti et al., 2004). Summarizing, these results support a clear dependence on mitochondrial functionality for the fertilizing capacity of human spermatozoa.

Despite all aforementioned, apoptosis must not be considered only as a fail on germ cell physiology or a secondary effect of certain pathologies and exogenous hazardous agents. Apoptosis orchestrates correct function and development of the male germ cell from the early embryonic stages of gonadal differentiation to the fertilization. In this report, we will highlight the roles played by apoptosis along the development of fetal germ line cells as a selective instrument that guarantees the delivery of genetic message to offspring.

## Primordial germ cells (PGC)

**Figure 1:**
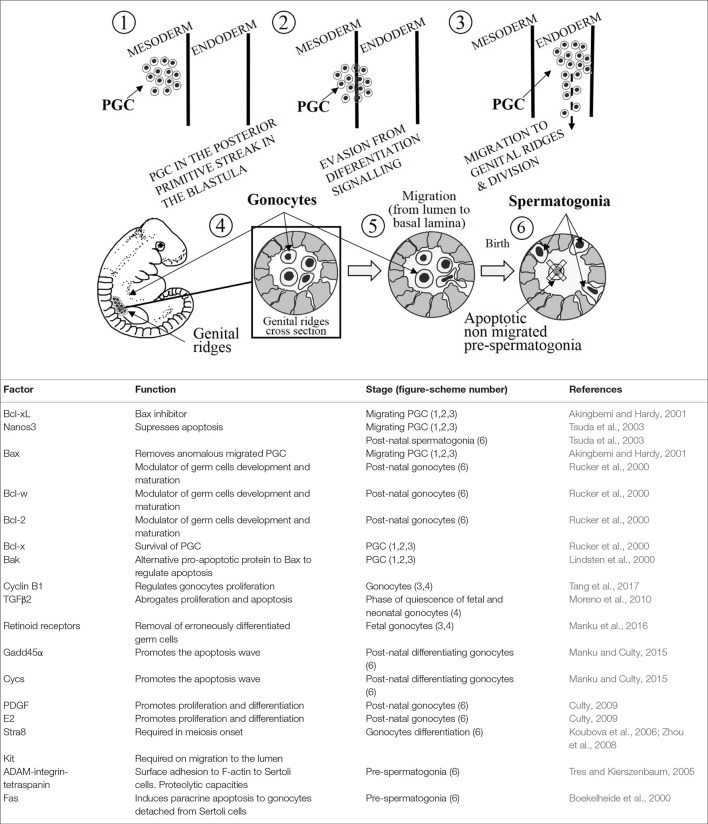
**(Upper)** A schematic illustration of development of germ cells. The numbers inside the circles are tagging the mentioned stages during the development. **(Lower)** The table summarizes the main apoptosis-related factors, their functions and the stages represented in the upper illustration (number in brackets).

The primordial germ cells (PGC) represent the most primitive cell type of the germ line in an organism that reproduces sexually. Their differentiation and development take place as an early event in the embryogenesis of mammals. PGC arise in the posterior primitive streak of the embryo, in the blastula. PGC migrate from the epiblast into an extraembryonic region, and subsequently, during early gastrulation PGC re-enter to reach gonadal ridges (developing gonads) by performing reiterative divisions along this migratory route (Richardson and Lehmann, 2010). Before gastrulation, the precursors of PGC are already committed and ready for migration. Such an early commitment and migration into an extraembryonic region allow germ cells to be isolated from the signaling, which induces somatic cell lineage differentiation (upper sub-Figure 1, stages 1,2,3) (De Felici, 2013). Apoptosis plays a selective role by removing cells carrying out an anomalous migration. During this period, the amount of PGC is submitted to the harmony between Bcl-xL and Bax proteins, which determines the death or survival process. Notwithstanding that, intrinsic apoptosis has been a pathway closely associated to cell damage (Akingbemi and Hardy, 2001; Bejarano et al., 2009), in this instance the excessive number of PGC is modulated by inducing the permeability of the mitochondrial membrane (Rucker et al., 2000). Thus, it is easy to understand that later experimental evidences reported that Bcl-x knockout mice exhibited severe defects in male germ cells during development (Kasai et al., 2003). Indeed, adult mice with two mutant *Bcl-x* alleles lacked spermatogonia and were sterile (Rucker et al., 2000). Although Bax, Bcl-w, and Bcl-2 are the major modulators of germ cell development and maturation after birth, Bcl-x is essential for the survival of PGC during early stages in the fetal gonad. Interestingly, the combination *Bcl-x* hypomorph with Bax^−/−^ reached a positive and alternative balance for these molecules in the development of PGC (Rucker et al., 2000).

*Nanos3*, is a gene that has been shown to be expressed in the murine PGC from their formation until their establishment into the gonads (upper sub-Figure 1, stages 1, 2, 3), and it is intriguingly re-expressed in post-natal testes (upper sub-Figure 1, stage 6) (Tsuda et al., 2003). *Nanos3* knockout mice are sterile and display gonadal atrophy (male and female). There is an absence of PGC in *Nanos3*^−/−^ murine embryos which is caused by the removal of migrating PGC during embryogenesis. And in point of fact, positive caspase-3 immunostaining has been reported in migrating PGC of *Nanos3*^−/−^ murine embryos (Suzuki et al., 2008). Suppression of the apoptotic Bax-dependent pathway entails the loss of migrating PGC is abrogated (Runyan et al., 2006). However, in double knockout mice lacking Bax and Nanos3, the sensitivity to apoptosis is attenuated compared with the single knockout Nanos3^−/−^ PGC.

More than likely, Bak could act as a mediator of the Bax-independent signaling, given that it is similar to Bax in terms of its structure and function. Although Bak single knockout mice do not show phenotypical changes, double knockout Bax^−/−^/Bak^−/−^ mice reveal more drastic variations in the phenotype comparing to Bax single knockout (Lindsten et al., 2000). These experimental findings, and the highly expressed Bak mRNA in migrating PGC (Runyan et al., 2006), indicate that Bak represents an alternative pathway to regulate the apoptosis in PGC (Lindsten et al., 2000). Summing up, apoptosis takes place affecting the mitochondrial membrane permeability, which plays a pivotal role in the modulation of apoptosis in PGC to avoid anomalous migration into the gonads (upper sub-Figure 1, stages 3, 4).

## Gonocytes

Gonocytes could be found at the fetal and neonatal stages before being differentiated into spermatogonial stem cells. During migration from extraembryonic region and the subsequent settlement into the gonads, PGC complete several rounds of proliferation to be finally differentiated into gonocytes within testes. After colonization, gonocytes continue proliferating concomitantly with a burst of apoptosis (upper sub-Figure 1, stages 4, 5) (Coucouvanis et al., 1993). The gonocytes proliferation is critically dependent on cyclin B1, what is essential for the correct spermatogenesis. In this line, experimental findings showed that the lack of cyclin B1 postnatal murine gonocytes entails the depletion of germ cells due to the mitotic arrest and increased apoptosis (Tang et al., 2017). Most gonocytes, but not all, undergoes an inhibition of proliferation as spermatogonial stem cells from 17-day post-conception, once the birth takes place they resume mitosis (Moreno et al., 2010). In this phase of quiescence none of gonocytes will die, however the proliferative fetal and post-natal gonocytes will do (Olaso and Habert, 2000). Transforming Growing Factor β (TGFβ) signaling is involved in the regulation of a vast number of germ cell-linked processes such as proliferation, differentiation and apoptosis from fetal germ cells to the adulthood spermatogenesis. TGFβ2 has been described as an autocrine down regulator of the proliferation and apoptosis during fetal and neonatal gonocytes (upper sub-Figure 1, stages 4, 5, 6). The abrogation of TGFβ signaling inhibits partially the phase of gonocyte quiescence, the massive proliferation of this germ stem cells will lead to a premature senescence of gonocytes by exhaustion. In other words, TGFβ signaling seems to have an essential role for a normal spermatogenesis (Moreno et al., 2010).

Retinoid acid (RA), which is the active metabolite of vitamin A, has been extensively investigated for ability to induce differentiation in great number of different cell types. RA is known to bind RA nuclear receptors as RAR α, β, and γ and retinoid receptors as RXR α, β, and γ, being essential for the fate of fetal germ cells (Bowles et al., 2006). RA was reported to exert a dual effect on proliferating fetal gonocytes (upper sub-Figure 1, stages 4, 5). Evidence indicated that RA induced a slight increase in proliferation on *ex-vivo* organ cultures mitotic gonocytes culture concomitantly with a wide increase of apoptosis leading a drop in the number of gonocytes (Livera et al., 2000). In addition, RA has been shown to prevent the decrease in cell proliferation of organ cultures, but not on gonocytes mitotically arrested where manifested an anti-differentiation effect (Trautmann et al., 2008). In line with these results, a study reported that co-culturing gonocytes and Sertoli cells, RA diminished the proliferation of gonocytes (Boulogne et al., 2003). Summarizing, RA prevents the mitotic arrest of fetal gonocytes, impeding their differentiation and inducing gonocyte apoptosis. In other words, RA promotes fetal gonocytes proliferation while increases apoptotic events. The most plausible hypothesis proposes that these apparently controversial dual effects on fetal gonocytes might be a physiological strategy for the removal of erroneously differentiated germ cells (Manku et al., 2016).

RA induces the expression of Stimulated by retinoic acid gene 8 (*Stra8*) and *Kit* mainly in undifferentiated spermatogonia. KIT has been recognized as a marker of differentiating spermatogonia and STR8 to be required prior the meiosis onset. These studies, carried out treating germ cells with RA in a free-somatic cell system indicate that Sertoli cells are likely involved in the modulation of gonocytes differentiation (upper sub-Figure 1, stages 4, 5, 6) (Koubova et al., 2006; Zhou et al., 2008). Likewise, Sertoli cells also secrete anti- and pro-apoptotic factors being likely the origin of the apoptotic signal in early postnatal germ cells. Subsequently, the pro-apoptotic factors (Fas) are completed by the phagocytic removal function shown by Sertoli cells (Boekelheide et al., 2000; Manku et al., 2016).

Intriguingly, growth arrest and DNA-damage-inducible-45-alpha (*Gadd45*α) is particularly expressed in germ cells. It has been also reported that, among several pro-apoptotic genes, *Gadd45*α expression is concomitantly upregulated with somatic cytochrome *c* (*Cycs*) in differentiating gonocytes (Manku and Culty, 2015). Inactivation of *Gadd45*α and *Cycs* expressions has been linked to uncontrolled proliferation, evasion of apoptosis and cancer proneness (Rosemary Siafakas and Richardson, 2009; Liebermann et al., 2011). Those results indicate that both genes might play a key role in differentiating gonocytes as well as in the first wave of germ cell apoptosis (Manku and Culty, 2015).

Sertoli cells synthesize and secrete in a paracrine fashion platelet derived growth factor (PDGF) and 17β-estradiol (E2), which have been shown to stimulate the proliferation of neonatal gonocytes in rat (Li H. et al., 1997). The simultaneous action of PDGF and E2 plays and activate role on neonatal gonocyte proliferation (Culty, 2009). Despite there is a predominance of PDGFR over c-kit, both proteins, and their phosphorylation by tyrosine kinase activities are involved in the modulation of apoptosis and proliferation in neonatal gonocytes (Basciani et al., 2008). E2, for its part, shows a double action on neonatal murine gonocytes as anti-mitotic and pro-apoptotic agent in mice with inactivated estrogen receptor β (ERβ) (Delbès et al., 2004). On the contrary, in organotypic cultures of rat testes, the treatment with exogenous E2 did not induce either a drop in the number of gonocytes or an increase in the ratio of proliferative gonocytes (Delbès et al., 2007). Moreover, despite E2 had pro-apoptotic effect on neonatal gonocytes, E2 exerts a pro-survival action on adult human germ cells (Pentikäinen et al., 2000). Putting together all these results, it seems that E2 involvement on modulation of mitosis is tightly linked to additional signaling mechanisms, therefore E2 might show different effects on germ cells depending on species, the mitotic activity and the cell stage.

## Birth, from gonocytes to spermatogonia

After birth, a relocation of gonocytes takes place from the lumen to the basement of testicular cords. On the one hand, this migration is thought to be required for the correct differentiation to spermatogonia; on the other hand, this migration aims to establish a tight interaction between Sertoli cells and pre-spermatogonia, which once this interaction is created pre-spermatogonia develop a strong dependence on it (Hutson et al., 2013). Pre-spermatogonia remaining in the center of the cord after migration are targeted to apoptosis (upper sub-Figure 1, stages 5, 6). Unknown still remains the regulatory factors involved in this differentiation process, although PDGF has been implicated (Basciani et al., 2008). ADAM-integrin-tetraspanin complex is a protein complex made up by integrins α3β1 and α6β1 and tetraspanins, it combines cell surface adhesion and proteolytic capacities. This complex is organized in a more sophisticate complex of membrane microdomains called the tetraspanin web (Tres and Kierszenbaum, 2005). F-actin binds tetraspanin web stabilizing the attachment of pre-spermatogonia to Sertoli cells, in such a way that the detachment of Sertoli cells leads to structural changes and fragmentation of F-actin within the pre-spermatogonium. Subsequently, Sertoli cells release locally soluble Fas-ligand which binds FasR in the detached pre-spermatogonium leading to extrinsic induced apoptosis, or even initiating a spontaneous Fas-independent apoptotic process (Tres and Kierszenbaum, 2005). On the other hand, gonocytes migrated in a timely manner and correctly relocated at the basement membrane evade the apoptotic signaling, likely they develop unresponsiveness in their new microenvironment (upper sub-Figure 1, stages 5, 6) (Sinha Hikim and Swerdloff, 1999; Manku et al., 2016). Thus, there is an active communication between germ cells and their support Sertoli cells. Sertoli cells, by inducing paracrine apoptosis, restrict the number of supported germ cells guaranteeing their correct physiology.

Before birth, testicles must descend into the scrotum. When an undescended testicle happens, in one or both, is called cryptorchidism and it should be surgically repaired. A study carried out in rat reported that, the effects cause by increased temperature due to undescended testes appear after germ cells transformation into spermatogonial sperm cells (SSC) (Zorgniotti, 1991). If cryptorchidism occurs, gonocytes should undergo apoptosis, the excess of persisting gonocytes are prone to carcinoma *in situ* (CIS) and cancer in cryptorchidism. Apoptosis is the reason why in some models of mice with cryptorchidism no germ cells were present in the testis, but also is a physiological strategy to avoid cancer in testes.

After birth gonocytes are differentiated into the mitotically quiescent type-A spermatogonia. Curiously, in human, after 1–2 year the germ cell amount is reduced in less than half comparing to birth total number (Hadziselimovic, 1983). No gonocytes remain by 2 years old, non-differentiated gonocytes are removed by apoptosis (Huff et al., 2001). However, in rat testis, in a frame of a week most of gonocytes are removed (Roosen-Runge and Leik, 2000). In mice and rats, it has been reported that, the first wave of spermatogenesis, which is concomitant with a burst of apoptosis, implicates caspase 2, caspase 3, 8, and 9 involving extrinsic and intrinsic pathway of apoptosis (Zheng et al., 2006; Moreno et al., 2010). By 3–4 years old in human type-A spermatogonia become type-B spermatogonia, and at that moment, they migrate into the center of testicular cord maturing to primary spermatocytes. Rat and mice represent a reliable system to study the mammal development and translating conclusion into humans. Although caution is taken regarding the length and the time when of events take place.

In healthy males, it has been quantified a degeneration of 75% germ cells as redundant from spermatogonia to mature spermatozoa (Rodriguez et al., 1997). Although, in general terms, the ensemble of apoptosis bursts, that takes place in different phases of germinal cell development, is the lead actor responsible of such a severe selection. Putting together the aforementioned studies, it seems clear that the more complete is the understanding of male germ cell development and spermatogenesis, the bigger is the potential of germ stem cells to be used for recolonizing the infertile testis (reviewed in Waheeb and Hofmann, 2011). We summarize that apoptosis is an essential mechanism for the suitable development of germ cells from the first embryonal stages to the complete spermatogenesis. The onset of apoptosis in right place of gonads, and at the right time is critical for assuring the regular fertility and the delivery of the perfect genetic message to the new organism.

## Author contributions

IB wrote the review. AR and JP critically discussed the scientific literature, revised the work and approved its version to be submitted.

### Conflict of interest statement

The authors declare that the research was conducted in the absence of any commercial or financial relationships that could be construed as a potential conflict of interest.

## Abbreviations

Cis: Carcinoma *in situ*
*Cycs*: somatic cytochrome *c*
E2: 17β-estradiol
ERβ: Oestrogen receptor beta
*Gadd45α*: Growth arrest and DNA-damage-inducible-45-alpha
MAC: Mitochondrial apoptosis-induced channel
PDGF: Platelet derived growth factor
PGC: Primordial germ cells
RA: Retinoid acid
RAR: Retinoid acid nuclear receptors.
RXR: Retinoid receptors
SSC: Spermatogonial sperm cells
Stra8: Stimulated by retinoic acid gene 8
TGFβ: Transforming Growing Factor β
ΔΨ_m_: Mitochondrial membrane potential.
